# The Effect of a Combined Intermittent Fasting Healthy Plate Intervention on Anthropometric Outcomes and Body Composition Among Adults With Overweight and Obesity: Nonrandomized Controlled Trial

**DOI:** 10.2196/51542

**Published:** 2024-04-10

**Authors:** Shazana Rifham Abdullah, Ruziana Mona Wan Mohd Zin, Nur Hayati Azizul, Nur Suffia Sulaiman, Norhayati Mustafa Khalid, Roshan Jahn Mohd Salim Mullahi Jahn, Muhamad Khairul Nazrin Khalil, Norhashimah Abu Seman, Nur Azlin Zainal Abidin, Azizan Ali, You Zhuan Tan, Azahadi Omar, Zamtira Seman, Abqariyah Yahya, Mohd Fairulnizal Md Noh

**Affiliations:** 1 Nutrition, Metabolism and Cardiovascular Research Centre, Institute for Medical Research National Institutes of Health, Ministry of Health Shah Alam Malaysia; 2 Sector for Biostatistic and Data Repository National Institutes of Health, Ministry of Health Shah Alam Malaysia; 3 Department of Social and Preventive Medicine, Faculty of Medicine Universiti Malaya Kuala Lumpur Malaysia

**Keywords:** intermittent fasting, dry fasting, healthy plate, obesity, overweight

## Abstract

**Background:**

Adult obesity and overweight pose a substantial risk to global public health and are associated with various noncommunicable diseases. Although intermittent fasting (IF) is increasingly used as a relatively new dietary strategy for weight loss, the effectiveness of 2 days per week of dry fasting remains unknown.

**Objective:**

This study aims to evaluate the effectiveness of a combined dry IF and healthy plate (IFHP) and healthy plate (HP) intervention in improving anthropometric outcomes and body composition.

**Methods:**

This nonrandomized controlled trial involved 177 adults who were overweight and obese. Among them, 91 (51.4%) were allocated to the IFHP group and 86 (48.6%) were allocated to the HP group. The overall study duration was 6 months (October 2020 to March 2021). The intervention was divided into 2 phases: supervised (3 months) and unsupervised (3 months). The data were collected at baseline, after the supervised phase (month 3), and after the unsupervised phase (month 6). Anthropometric (weight, height, waist circumference, and hip circumference) and body composition (body fat percentage, body fat mass, skeletal muscle mass, and visceral fat area) data were measured at all 3 data collection points. Sociodemographic data were obtained using a questionnaire at baseline.

**Results:**

Most participants were female (147/177, 83.1%) and Malay (141/177, 79.7%). After 3 months, there were significant reductions in weight (difference −1.68; *P*<.001), BMI (difference −0.62; *P*<.001), body fat percentage (difference −0.921; *P*<.001), body fat mass (difference −1.28; *P*<.001), and visceral fat area (difference −4.227; *P*=.008) in the IFHP group, whereas no significant changes were observed in the HP group. Compared to baseline, participants in the IFHP group showed a significant decrease in weight (difference −1.428; *P*=.003), BMI (difference −0.522; *P*=.005), body fat percentage (difference −1.591; *P*<.001), body fat mass (difference −1.501; *P*<.001), visceral fat area (difference −7.130; *P*<.001), waist circumference (difference −2.304; *P*=.001), and hip circumference (difference −1.908; *P*=.002) at month 6. During the unsupervised phase, waist (IFHP difference −3.206; *P*<.001, HP difference −2.675; *P*=.004) and hip (IFHP difference −2.443; *P*<.001; HP difference −2.896; *P*<.001) circumferences were significantly reduced in both groups (*P*<.01), whereas skeletal muscle mass (difference 0.208; *P*=.04) and visceral fat area (difference −2.903; *P*=.003) were significantly improved in the IFHP group only. No significant difference in the between-group comparison was detected throughout the intervention (all *P*>.05).

**Conclusions:**

A combined IFHP intervention was effective in improving anthropometric outcomes and body composition in adults with overweight and obesity.

**International Registered Report Identifier (IRRID):**

RR2-10.2196/33801.

## Introduction

### Background

Obesity, defined as a disease by the American Medical Association in June 2013 [1], poses greater and long-lasting harm to the collective health of adults and children worldwide. Despite being prioritized in health policies for decades, the prevalence of obesity continues to rise across countries and socioeconomic statuses. Globally, the obesity rate has almost tripled since 1975 with 39% and 13% of adults being overweight and obese, respectively [2]. A similar pattern has been observed in Malaysia where the prevalence of overweight and obesity in adults had increased by 12.6% from 2011 to 2019 [3]. In 2019, the National Health and Morbidity Survey reported that half of the adult population in Malaysia was either overweight (30.4%) or obese (19.7%) compared with 29.4% and 15.1% in 2011, respectively [3].

Obesity is a leading health problem that increases the risk of multiple health conditions such as cardiovascular diseases, type 2 diabetes mellitus, dyslipidemia, asthma, infertility, and certain types of cancers. In addition, it has adverse effects on the economy and national productivity. In 2019, the economic impact of overweight and obesity across 161 countries was estimated to be an average of 2.19% of the gross domestic product. If the current trends continue, it is projected to rise to 3.29% of the gross domestic product by 2060 [4]. Obesity has other associated indirect costs and losses, such as lost workdays, economic burdens stemming from premature mortality, and reduced work productivity [4].

Dietary modification is a keystone of weight management. As the foundation of weight loss, a state of negative balance must be achieved. Over the decades, there has been much debate on how to attain and maintain this negative energy balance by researching the most practical and feasible methods for weight loss. Studies have demonstrated that different dietary regimes are not only effective in lowering weight but also in improving the risks of chronic diseases such as type 2 diabetes mellitus, hypertension, and nonalcoholic fatty liver disease [5-8]. Intermittent fasting (IF) is a form of calorie restriction that consists of various eating plans that cycle between fasting and nonfasting states over a defined period. The effectiveness of wet IF in reducing weight and improving cardiometabolic effects has been proven in recent studies [9,10]. However, the benefits of dry fasting (except for Ramadan fasting) are not well documented. Wet IF is a form of fasting that restricts the consumption of all types of food and drink except water [11], whereas dry IF is defined as a complete fast in which no food or fluid intake is allowed [12].

A practical and easy method for restricting calorie intake is by controlling the portion size of a meal. This method has been widely practiced worldwide and differs based on the culture and eating habits [13,14]. In Malaysia, the Malaysian Healthy Plate is a portion control method that was created to translate the messages in the Malaysian Dietary Guidelines 2010 and Malaysian Food Pyramid 2010 [15]. It is a single-meal guide that suggests food intake following the quarter-quarter-half concept, which divides the plate into a quarter plate of grains or grain products; a quarter plate of fish, poultry, meat, or egg; and a half plate of fruits and vegetables [16].

### Objectives

Although numerous studies have proven the effectiveness of conventional IF in reducing body weight [17], the efficacy of 2 nonconservative days per week of dry IF remains unclear. Furthermore, the reported benefits of the Malaysian Healthy Plate on improving health are still lacking, despite being widely practiced in Malaysia since 2016. To date, no other study has reported the effects of combined IF and a healthy plate (HP) dietary protocol on health. Hence, this nonrandomized controlled trial aims to determine the effectiveness of combined IF and HP (IFHP) and HP interventions in improving anthropometric outcomes and body composition. We hypothesized that there would be a significant improvement in the outcomes in both groups, with more prominent changes observed in the IFHP group because of the added IF intervention.

## Methods

### Study Population and Design

This nonrandomized controlled study involved a total of 177 participants with overweight or obesity. The participants were divided into 2 intervention groups, the combined IFHP group and the HP group, based on their designated workplace (the National Institutes of Health in Setia Alam; the Institute for Medical Research, Jalan Pahang; and the Institut Latihan Kementerian Kesihatan Malaysia [Teknologi Makmal Perubatan], Jalan Pahang). The distance between Jalan Pahang and Setia Alam is approximately 40 km. The allocation was conducted in a manner that aimed to prevent contamination bias and was chosen based on the practicality of monitoring the participants. The study population exhibited a rather homogeneous set of sociodemographic characteristics, environmental conditions, facility settings, and job types.

This study enrolled participants aged 19 to 59 years with a BMI ≥23 kg/m^2^ (overweight or obese) who were ready to participate in the intervention (assessed through readiness to participate in screening) and provided informed consent. The exclusion criteria included the following: (1) a recent involvement in weight loss programs (eg, IF, diet changes, physical activity changes, or activities that were performed constantly to reduce weight); (2) having any eating disorder; (3) diagnosed with diabetes and hypertension (on medication) or other metabolic health disturbance conditions; (4) taking any medication or supplements that can affect the study outcomes; (5) pregnancy; and (6) a lack of capacity or language skills to independently follow the protocol. A detailed description of the study design and methodology has been published elsewhere [18].

### Sample Size Calculation

Sample size calculation was conducted using the PS: Power and Sample Size Calculation program (version 3.1.6; William D Dupont and Walton D Plummer, Jr). The sample size was calculated based on a between-group mean difference (δ) of 5% (SD 10%) in weight loss, 95% CI (α=.05), and a power of study of 80% (1−β=.80). According to a review by Ryan and Yockey [19], a minimum weight loss of 5% is needed to improve cardiometabolic risk such as hypertension, diabetes mellitus, and hyperlipidemia. The minimum sample size required for this study was 64 participants for each arm. After considering 40% attrition, the calculated sample size for each arm was 90 participants, and a total of 180 participants were required for this study.

### Dietary Protocol

Participants in the combined IFHP group were required to observe dry fasting from dawn to dusk for 2 days a week (Mondays and Thursdays) and practice HP for the rest of the week. During the fasting days, they were encouraged to have a meal before dawn, and no food or drink was allowed following that meal (approximately 13 hours) until sunset. Smoking and sexual activity were also forbidden during the fasting day, following the Islamic fasting obligation. For the remaining 5 days of the week, they were asked to apply the HP concept while consuming meals.

In the HP group, the participants were required to practice the HP concept daily, which entailed the division of plate portions into a quarter for protein, a quarter for complex carbohydrates, and a half for fruits and vegetables. Although they were advised to practice the HP concept for all 3 main meals per day, compliance with the dietary protocol was considered if they applied the concept to at least 1 main meal per day. The adherence to dietary protocols was monitored by trained research assistants through a daily record of food intake picture (1 meal per day) and a weekly fasting record.

### Study Procedures

There were two stages of the recruitment phase: (1) health screening and (2) readiness to participate in screening. Those who were interested in joining the study were screened for the inclusion and exclusion criteria by the study team. Eligible volunteers were screened for their readiness to participate in the study, namely their motivation, enthusiasm, and willingness to commit to study protocols. Only participants who were ready to commit were included in the study.

The overall duration of the intervention was 6 months, which included 3 months of a supervised phase and another 3 months of an unsupervised phase. During the supervised phase, a reminder to fast was sent to their mobile phone on the eve of fasting days and compliance was recorded twice weekly. For HP, all participants were required to send at least 1 picture of their meal every day to the research assistants. On the contrary, no fasting reminder or meal pictures were exchanged during the unsupervised phase.

The data collection was conducted at 3 time points: baseline (before the intervention started), month 3 (at the end of the supervised phase), and month 6 (at the end of the unsupervised phase). During baseline data collection, the participants were asked to answer questions on sociodemographic factors. Anthropometric measurements were taken, including body composition analysis, at all 3 points of data collection.

Measurements of body weight and height were conducted using a Seca electronic column scale (Seca GmbH and Co KG) in kg and cm to the nearest 0.1 kg and 0.1 cm, respectively. Body weight was measured in light clothing and participants were required to remove their outer garments and shoes. BMI was calculated by dividing weight by height squared (kg/m^2^) and categorized into overweight (23.0-27.4 kg/m^2^), obese I (27.5-32.4 kg/m^2^), obese II (32.5-37.4 kg/m^2^), and obese III (≥37.5 kg/m^2^) based on the cutoff points for public health action in Malaysia [20]. Waist and hip circumferences were measured using a Seca measuring tape (Seca GmbH and Co KG) to the nearest 0.1 cm, with the participants standing. Waist circumference was measured at the midpoint between the top of the iliac crest and the lower margin of the last palpable rib, whereas hip circumference was measured at the widest diameter around the buttocks. To minimize measurement error, 2 measurements were taken for each parameter and the average was calculated. The waist-to-hip ratio was calculated by dividing the waist measurement by the hip measurement.

For body composition analysis, parameters such as body fat percentage, body fat mass, muscle mass, and visceral fat area were measured. These parameters were measured using a tetrapolar bioimpedance multifrequency InBody 770 analyzer (Biospace). Personal profiles (age, height, weight, and sex) were entered after reading the measurement. Before the measurement, the participants were asked to remove all metal items and stand barefoot on the device in a supine position. They then grasped the handles of the units with their thumbs and palms while maintaining direct contact with the electrode. In total, 8 polar tactile electrodes were used in the bioelectrical impedance analysis (BIA): 2 for each thumb, 2 for the palms, and 2 for the front and 2 for the back of each foot. The body composition results were computed using proprietary prediction algorithms that were built into the firmware of the device and applied only to the device being studied. The total time required for the measurement was approximately 2 minutes [21].

The International Physical Activity Questionnaire (IPAQ)–Short Form was used to assess the physical activity of the participants over the past week. The questionnaire has been validated for use among adults in 12 countries [22]. In this study, we used the Malay version of the IPAQ-Short Form, which was validated using the data from the 2011 National Health and Morbidity Survey [23]. The participants were asked to record the number of days in the previous week that they engaged in specific activities (vigorous and moderate activities and walking) for at least 10 minutes as well as the amount of time (in min) that they engaged in a particular activity on an average day. Energy expenditure or metabolic equivalent of task (MET) minutes per week was used to determine the physical activity based on the IPAQ scoring protocol. To calculate the MET scores for each activity, the total minutes spent on vigorous activity, moderate activity, and walking over the past week were multiplied by 8, 4, and 3.3, respectively. The total physical activity score was calculated as the sum of the MET scores for each of the 3 activity categories. All participants were asked to maintain their usual physical activity throughout the study period. The physical activity score was included as one of the controlled variables in the analysis.

The Food Frequency Questionnaire (FFQ) was used to measure the participants’ dietary intake over the previous month. The questionnaire consisted of questions covering the frequency of consuming cereals and cereal products, fast food, meat and meat products, fish and seafood, eggs, legumes and legume products, milk and milk products, vegetables, fruits, drinks, alcoholic drinks, confectionaries, bread spreads, and flavor intake. We used the validated Malay version of the FFQ, which consists of 165 items [24], and the participants required approximately 30 minutes to answer the questions at each point of data collection. We calculated the calorie intake using nutrient data that were extracted from the Nutritionist Pro nutrition analysis software (version 7.8.0; Axxya Systems). The calorie intake changes observed in this study confirmed the dietary protocol adherence.

### Statistical Analyses

Statistical analysis was performed using the SPSS software (version 25; IBM Corp). For continuous variables, the normality of distribution was tested using the Kolmogorov-Smirnov test. Normally distributed data were presented as mean (SD), whereas the median (IQR) summarized the skewed data. For categorical variables, frequencies were calculated and presented as percentages. All variables were compared using the independent 2-tailed *t* test or Mann-Whitney *U* test for continuous variables and the chi-square or Fisher exact test (n≤5 in any cell) for categorical variables. To determine the changes in outcome following the intervention, a generalized estimating equation (GEE) analysis was performed, which was adjusted for possible confounders such as age, ethnicity, sex, and physical activity. The GEE statistical analysis has been widely used owing to its robustness and ability to analyze correlated, nonnormally distributed data [25]. Compared with the repeated measures ANOVA, GEE provides more flexibility in handling missing data and accommodates unbalanced designs. The time effect was analyzed for all 3 comparisons: baseline versus month 3, baseline versus month 6, and month 3 versus month 6. To control for the overall type I error rate, we adjusted for multiple comparisons using the Bonferroni correction method in the GEE analysis. For the calorie intake analysis, we excluded participants with extreme calorie intake (<500 and >3500 kcal/day for women and <800 and >4000 kcal/day for men) [26]. All statistical tests were 2-sided, and the significance level was set at .05.

### Ethical Considerations

This study was approved by the Medical Research and Ethics Committee, Ministry of Health Malaysia (NMRR-19-3261-51726) and was conducted in full conformity with the current revision of the Declaration of Helsinki and the International Council for Harmonisation Guidelines for Good Clinical Practice. Before recruitment, all participants were thoroughly briefed about the potential risks involved with participating in this study, and written informed consent was obtained from them. The participants had the autonomy to discontinue their involvement in the study at any point. The data were treated as strictly confidential, and each participant was assigned a unique anonymous identifier. Each participant received a reimbursement of RM30 (US $6) after each data collection, resulting in a total compensation of RM60 (US $13) per participant across the study period. This study was registered with ClinicalTrials.gov (NCT05034653).

## Results

### Flow of Study Participants

**Figure 1:**
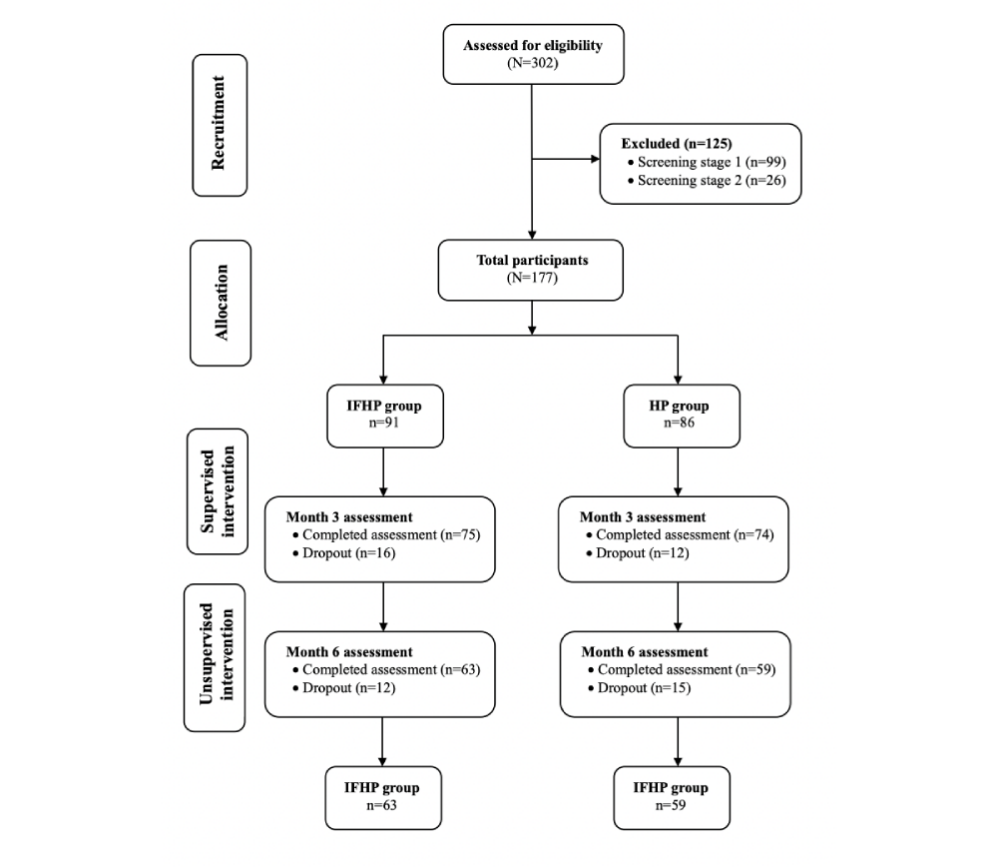
Flowchart of participants through each stage of the study. HP: healthy plate; IFHP: intermittent fasting healthy plate.

Overall, 302 volunteers were interested in joining the study, of which 99 (32.8%) were excluded because of the failure to meet the eligibility criteria during the first screening stage. Another 27 volunteers were excluded during the screening process in stage 2 for multiple reasons, such as pursuing further education, commitment issues, or relocating. Hence, a total of 177 participants were recruited in this study: 91 (51.4%) in the IFHP group and 86 (48.6%) in the HP group. During the supervised phase of the intervention, a total of 28 participants withdrew from the study (IFHP: 16/28, 57%; HP: 12/28, 43%), whereas 27 participants withdrew during the unsupervised phase (IFHP: 12/27, 44%; HP: 15/27, 56%). The reasons for dropout included the inability to commit to the study intervention (24/55, 44%); pregnancy (13/55, 24%); transfer to a different workplace (8/55, 15%); started medication for hypertension, diabetes, or hypercholesterolemia (5/55, 9%); and other reasons (5/55, 9%). Finally, 63 and 59 participants completed the study in the IFHP and HP groups, respectively (Figure 1).

### Sociodemographic Characteristics

Table 1 summarizes the characteristics of the participants based on the intervention groups at baseline. A majority of the participants were female (147/177, 83.1%) and Malay (141/177, 79.6%) with a mean age of 34.47 (SD 7.40) years. Most participants (121/177, 68.4%) had a diploma or degree as the highest educational status. In terms of background illness history, 5.1% (9/177) and 1.1% (2/177) of the participants reported being diagnosed with hyperlipidemia and hypertension, respectively. However, none of the participants were taking any medication for the illnesses. Participants in the IFHP group had a higher mean weight (72.50, SD 18.55 kg) than those in the HP group (70.60, SD 16.28 kg); however, this difference was not statistically significant (*P*=.13). In addition, no significant differences in physical activity, daily calorie intake, other body composition, and anthropometric parameters were found between the participants from both intervention groups (Table 1). Detailed *t* test results are presented in Multimedia Appendix 1.

**Table 1:** Baseline characteristics of the participants based on the intervention groups.

Characteristics				Total (N=177)		Intervention groups				*P* value
						IFHP^a^ (n=91)		HP^b^ (n=86)		
**Sociodemographics**										
	**Sex, n (%)**									.32^c^
		Male	30 (16.9)		18 (19.8)		12 (14)			
		Female	147 (83.1)		73 (80.2)		74 (86)			
	**Ethnicity, n (%)**									.02^c^
		Chinese	4 (2.3)		0 (0)		4 (4.7)			
		Indian	12 (6.8)		4 (4.4)		8 (9.3)			
		Malay	141 (79.6)		80 (87.9)		61 (70.9)			
		Others	20 (11.3)		7 (7.7)		13 (15.1)			
	Age (y), mean (SD)			34.47 (7.40)		33.82 (7.50)		35.15 (7.27)		.23^d^
	**Highest education status, n (%)**									.95^c^
		Secondary school	24 (13.6)		13 (14.3)		11 (12.8)			
		Diploma or degree	121 (68.4)		62 (68.1)		59 (68.6)			
		Master or PhD	32 (18.1)		16 (17.6)		16 (18.6)			
	**Background illness, n (%)**									
		Hypertension	2 (1.1)		1 (1.1)		1 (1.1)		.74^c^	
		Hyperlipidemia	9 (5.1)		6 (6.6)		3 (3.5)		.5^c^	
		Heart disease	1 (0.6)		0 (0)		1 (1.2)		.49^c^	
		Others	1 (0.6)		0 (0)		1 (1.2)		.49^c^	
	Physical activity (MET^e^-min/wk), median (IQR)			829.50 (769.48)		706 (1055.70)		627 (629.50)		.63^f^
	Calorie intake (kcal/d), mean (SD)			2093.68 (645.08)		2082.03 (667.01)		2106.14 (625.21)		.82^d^
**Anthropometrics**										
	Body weight (kg), median (IQR)			71.70 (18.70)		72.50 (18.55)		70.60 (16.28)		.13^f^
	Height (cm), mean (SD)			158.36 (7.09)		158.67 (7.13)		158.04 (7.08)		.56^d^
	BMI (kg/m^2^), median (IQR)			28.43 (6.50)		28.48 (7.25)		28.39 (5.70)		.18^f^
	**BMI category, n (%)**									.51^c^
		Overweight	63 (35.6)		32 (35.2)		31 (36)			
		Obese class I	69 (39.0)		32 (35.2)		37 (43)			
		Obese class II	32 (18.1)		20 (22.0)		12 (14)			
		Obese class III	13 (7.3)		7 (7.6)		6 (7)			
	Waist circumference (cm), mean (SD)			92.46 (10.88)		93.49 (10.70)		91.36 (11.03)		.2^d^
	Hip circumference (cm), mean (SD)			108.86 (8.66)		109.39 (9.02)		108.31 (8.29)		.14^d^
	**Body composition**									
		Body fat percentage (%), mean (SD)	44.09 (6.17)		44.21 (6.36)		43.96 (6)		.79^d^	
		Body fat mass (kg), mean (SD)	33.26 (8.96)		34.08 (9.37)		32.37 (8.45)		.21^d^	
		Skeletal muscle mass (kg), mean (SD)	22.71 (4.90)		23.36 (4.80)		22.13 (4.96)		.13^d^	
		Visceral fat area (cm^2^), mean (SD)	166.52 (40.44)		168.13 (41.32)		164.77 (39.62)		.59^d^	

^a^IFHP: intermittent fasting healthy plate.

^b^HP: healthy plate.

^c^*P* values derived from the Pearson chi-square test.

^d^*P* values derived from the independent *t* test.

^e^MET: metabolic equivalent of task.

^f^*P* values derived from the Mann-Whitney *U* test (nonnormally distributed data).

### Changes in Anthropometric and Body Composition

#### Baseline Versus Month 3

Within the IFHP group, there was a significant reduction in weight (estimated margin [EM] mean difference −1.680; *P*<.001), BMI (EM mean difference −0.620; *P*<.001), body fat percentage (EM mean difference −0.921; *P*<.001), body fat mass (EM mean difference −1.280; *P*<.001), and visceral fat area (EM mean difference −4.227; *P*=.008) at month 3 compared to baseline. Meanwhile, no significant changes were observed in skeletal muscle mass (*P*=.43), waist circumference (*P*=.45), and hip circumference (*P*=.80; Table 2).

**Table 2:** Comparison of the anthropometric and body composition changes between the intermittent fasting healthy plate (IFHP) and healthy plate (HP) groups at baseline, month 3, and month 6.

Characteristics		Time				Time effect				Group effect	
		Baseline, mean (SE)	Month 3, mean (SE)	Month 6, mean (SE)	Baseline vs month 3, EM^a^ mean difference (*P* value)		Baseline vs month 6, EM mean difference (*P* value)	Month 3 vs month 6, EM mean difference (*P* value)	EM mean difference (*P* value)		
**Weight (kg)**											1.274 (.55)
	IFHP	78.83 (1.96)	77.15 (1.96)	77.4 (1.99)	−1.68 (<.001)		−1.428 (.003)	0.252 (.73)			
	HP	76.96 (2.55)	76.64 (2.57)	76.83 (2.55)	−0.317 (.49)		−0.133 (>.99)	0.184 (>.99)			
**BMI (kg/m^2^)**											0.473 (.52)
	IFHP	29.45 (0.64)	28.83 (0.66)	28.93 (0.67)	−0.62 (<.001)		−0.522 (.005)	0.098 (.08)			
	HP	28.69 (0.7)	28.59 (0.7)	28.66 (0.7)	−0.101 (.78)		−0.023 (>.99)	0.079 (>.99)			
**Body fat percentage (%)**											−0.062 (.93)
	IFHP	39.7 (0.72)	38.78 (0.75)	38.11 (0.74)	−0.921 (<.001)		−1.591 (<.001)	−0.67 (.07)			
	HP	39.57 (0.97)	39.4 (0.98)	39.39 (1.01)	−0.168 (.98)		−0.175 (>.99)	−0.008 (>.99)			
**Body fat mass (kg)**											0.534 (.71)
	IFHP	31.38 (1.36)	30.1 (1.37)	29.88 (1.37)	−1.28 (<.001)		−1.501 (<.001)	−0.22 (.64)			
	HP	30.58 (1.5)	30.37 (1.52)	30.51 (1.51)	−0.206 (.78)		−0.072 (>.99)	0.134 (>.99)			
**Skeletal muscle mass (kg)**											0.491 (.32)
	IFHP	26.34 (0.4)	26.2 (0.41)	26.41 (0.42)	−0.144 (.43)		0.064 (>.99)	0.208 (.04)			
	HP	25.62 (0.87)	25.77 (0.86)	25.79 (0.86)	0.151 (.98)		0.171 (>.99)	0.020 (>.99)			
**Visceral fat area (cm^2^)**											−0.431 (.94)
	IFHP	148.88 (6.29)	144.65 (6.28)	141.75 (6.23)	−4.227 (.008)		−7.13 (<.001)	−2.903 (.003)			
	HP	148.51 (7.41)	148.39 (7.45)	147.92 (7.49)	−0.118 (>.99)		−0.597 (>.99)	−0.479 (>.99)			
**Waist circumference (cm)**											1.398 (.40)
	IFHP	98.14 (1.56)	99.05 (1.57)	95.84 (1.60)	0.902 (.45)		−2.304 (.001)	−3.206 (<.001)			
	HP	93.9 (1.85)	94.72 (1.95)	92.05 (2.04)	0.823 (.45)		−1.852 (.10)	−2.675 (.004)			
**Hip circumference (cm)**											−0.212 (.88)
	IFHP	109.13 (1.3)	109.66 (1.32)	107.22 (1.32)	0.535 (.80)		−1.908 (.002)	−2.443 (<.001)			
	HP	106.91 (1.48)	108.77 (1.53)	105.88 (1.49)	1.861 (<.001)		−1.034 (.03)	−2.896 (<.001)			

^a^EM: estimated margin.

*P* values <.05 are considered statistically significant. *P* values were derived from the GEE test adjusted for sex, age, ethnicity, and physical activity.

In the HP group, there was a reduction in weight (EM mean difference −0.317; *P*=.49), BMI (EM mean difference −0.101; *P*=.78), body fat percentage (EM mean difference −0.17; *P*=.98), body fat mass (EM mean difference −0.206; *P*=.78), and visceral fat area (EM mean difference −0.118; *P*>.99). However, these changes were not statistically significant. The hip circumference significantly increased at month 3 compared to baseline (EM mean difference 1.861; *P*<.001).

There were no significant changes in other parameters (all *P*>.05; Table 2).

#### Baseline Versus Month 6

After 6 months, participants in the IFHP group showed significant reduction in weight (EM mean difference −1.428; *P*=.003), BMI (EM mean difference −0.522; *P*=.005), body fat percentage (EM mean difference −1.591; *P*<.001), body fat mass (EM mean difference −1.501; *P*<.001), visceral fat area (EM mean difference −7.130; *P*<.001), waist circumference (EM mean difference −2.304; *P*=.001), and hip circumference (EM mean difference −1.908; *P*=.002). However, there were no significant changes observed in skeletal muscle mass (*P*>.99; Table 2).

Moreover, hip circumference (EM mean difference −1.034; *P*=.03) showed a significant reduction among participants in the HP group after 6 months. Although there was a reduction in weight, BMI, body fat percentage, body fat mass, and visceral fat area, it was not statistically significant (all *P*>.99). The increase in skeletal muscle mass was also not statistically significant (*P*>.99; Table 2).

#### Month 3 Versus Month 6

Comparing month 6 to month 3, there were significant improvements in skeletal muscle mass (EM mean difference 0.208; *P*=.04), visceral fat area (EM mean difference −2.903; *P*=.003), waist circumference (EM mean difference −3.206; *P*<.001), and hip circumference (EM mean difference −2.443; *P*<.001) in the IFHP group. Otherwise, there were no significant changes in other parameters (all *P*>.05; Table 2).

Furthermore, participants in the HP group showed a significant reduction in waist circumference (EM mean difference −2.675; *P*=.004) and hip circumference (EM mean difference −2.896; *P*<.001) at month 6 compared to month 3. However, no other significant changes were observed (all *P*>.05; Table 2).

In view of the between-group comparison, there were no significant differences between the IFHP and HP groups observed at all time points (all *P*>.05; Table 2).

### Calorie Intake Changes

There was a significant reduction in calorie intake at month 3 and month 6 compared to baseline in both intervention groups (Table 3). Compared to baseline, participants in the IFHP group consumed an average of 325 kcal and 266 kcal fewer calories per day after 3 months (EM mean difference −325.09; *P*=.002) and 6 months (EM mean difference −266.70; *P*=.006), respectively. Similarly, there was a significant reduction in calorie intake at month 3 (EM mean difference −320.11; *P*=.004) and month 6 (EM mean difference −437.79; *P*<.001) compared to baseline in the HP group. However, there were no significant changes in calorie intake between month 3 and month 6 in both the IFHP (*P*>.99) and HP (*P*=.73) groups. There was also no significant difference in the between-group comparison throughout the intervention (*P*=.94; Table 3).

**Table 3:** Changes in calorie intake in intermittent fasting healthy plate (IFHP) and healthy plate (HP) groups at baseline, month 3, and month 6.

		Intervention groups	
		IFHP	HP
**Calorie intake (kcal/d), mean (SE)**			
	Baseline	2212.58 (105.49)	2166.54 (124.32)
	Month 3	1887.49 (120.85)	1846.43 (132.23)
	Month 6	1945.88 (114.46)	1728.75 (142.26)
**Time effect, EM^a^ mean difference (*P* value)**			
	Baseline vs month 3	−*325.09 (.002)^b^*	−*320.11 (.004)*
	Baseline vs month 6	−*266.7 (.006)*	−*437.79 (<.001)*
	Month 3 vs month 6	58.4 (>.99)	−117.68 (.73)
Group effect, EM mean difference (*P* value)		−6.605 (.94)	−6.605 (.94)

^a^EM: estimated margin.

^b^Values indicate statistical significance.

## Discussion

### Principal Findings

To our knowledge, this is the only study to investigate the effect of IFHP regimens on anthropometric outcomes and body composition worldwide. This study found that a combined IFHP diet had a notable effect on weight, BMI, body fat percentage, body fat mass, and visceral fat area after 3 and 6 months of intervention. Although there were no changes in weight and BMI in the IFHP group at month 6 compared to those at month 3, visceral fat area was greatly reduced, whereas skeletal muscle mass showed a substantial improvement. After 6 months, a notable reduction in hip and waist circumference was observed among participants in both groups. The improvement was seen during the unsupervised period as there were no notable changes in these parameters at month 3. This study showed mixed findings when comparing supervised and unsupervised phases. After completing the supervised phase at month 3, the IFHP group showed substantial reductions in weight, BMI, body fat percentage, body fat mass, and visceral fat area, but not in skeletal muscle mass, waist circumference, or hip circumference. Meanwhile, only skeletal muscle mass, waist circumference, hip circumference, and visceral fat area improved significantly during the unsupervised phase. Overall, there was no notable difference between groups throughout the study period.

### Comparisons With Previous Works, Interpretations, and Implications

The short- and long-term effectiveness of the 2-day per week fasting in reducing weight has been reported in various studies [27-30]. Generally, the concept of a 5:2 IF diet is defined in most studies as restricting calorie intake to approximately 25% of the baseline energy intake twice a week, which makes it slightly different from our IF protocol. Furthermore, any zero-calorie beverages, such as plain water, were allowed during the fasting period. In 2013, a randomized controlled study was conducted to determine the efficacy of combined fasting (Islamic Sunnah Fasting for 2 days per week) and calorie restriction (reduction of 300-500 kcal/day from habitual energy intake) among healthy older men for 3 months. The study reported a significant interaction effect in body weight, BMI, fat percentage, fat mass, and fat-free mass after 3 months [31].

Studies reported in a recent systematic review and meta-analysis showed a positive effect of a portion control diet on weight and body composition [32]. The concept of the *plate model*, which was first introduced by the Community Nutrition Group of the British Dietetic Association and the Swedish Diabetic Association, provides a simple and practical visual tool to demonstrate the healthy portion of a meal intake [33]. Although the division and type of food in a meal differ according to culture and eating habits, the foundation concepts of a “plate model” or HP are calorie restriction and healthy redistribution of macronutrients, such as carbohydrates, proteins, and fibers. Our study found that among those on the HP diet alone, there were reductions in weight, BMI, body fat percentage, body fat mass, and visceral fat area after 3 and 6 months, but the changes were not statistically significant. This suggests that the addition of the IF diet 2 days per week to the HP diet had a greater impact on weight loss and improvement in body composition.

Notably, there was >4 cm^2^ and 7 cm^2^ loss of visceral fat area among participants in the IFHP group after 3 and 6 months, respectively. Our findings are in agreement with those of previous studies [34,35]. In a study conducted among overweight older men, there was a significant reduction in the visceral fat area following a 6-week time-restricted feeding intervention [35]. The role of fasting in facilitating lipolysis has been studied extensively. Fasting stimulates intracellular lipolysis, which is triggered by several hormonal changes such as decreased levels of insulin and increased cortisol, catecholamines, and growth hormone levels. Lipolysis is also stimulated by the sympathetic innervation of the adipose tissue [36]. A classical study supported the role of these hormones in triggering the lipolysis of adipose tissue following fasting by reporting that hypophysectomy or adrenalectomy in rats caused a reduction in plasma nonesterified fatty acid levels [37].

Central adiposity, represented by abnormal waist circumference, waist-to-hip ratio, and waist-to-height ratio, is highly associated with a higher risk of noncommunicable diseases such as cardiovascular disease and cancer [38-40]. Although both intervention groups showed significant reductions in waist and hip circumference after 6 months, greater mean differences were observed among participants in the IFHP group. Previous studies reported a similar significant reduction in waist and hip circumference after IF [34,41,42]. The London Ramadan Study is an observational study conducted during Ramadan to explore the health consequences of Ramadan IF. Besides other findings, this study showed a significant decrease in waist (mean difference −1.93 cm; *P*<.01) and hip circumference measurements (mean difference −2.86 cm; *P*<.01) [41]. In the IFHP group, we observed a significant decrease in both parameters during the unsupervised phase of the intervention (from month 3 to month 6). Although there was an insignificant increase in weight and BMI during this period, body fat percentage and visceral fat area were significantly reduced, which may explain the improvement in waist and hip measurements.

Apart from the weekly fasting record and daily meal picture, the reduction in calorie intake served as one of the adherence monitoring methods for the dietary protocols used in our study. The significant reduction in calorie intake at months 3 and 6 explained the improvement observed in the anthropometric outcomes and body composition parameters, especially among participants in the IFHP group. The findings also confirmed the principle of calorie restriction in IF and HP without the need to properly count the calorie intake allowable to be consumed per day to cause weight loss. A recent study reported no significant difference between time-restricted feeding and calorie restriction interventions in calorie reduction and weight loss after 3 months [43]. This finding provides an advantage to both interventions as they are more practical and easier to comply with.

Improvements in clinical end points and cardiometabolic biomarkers following changes in body composition have been reported in various studies [44,45]. However, for weight changes, it has been suggested that different degrees of weight loss contribute to different biomarker responses [46]. On the basis of the findings of a randomized controlled trial conducted among obese Americans, a 5% weight loss significantly improved the plasma concentrations of some cardiometabolic risk factor parameters, such as insulin, triglyceride, glucose, leptin, and alanine transaminase, but did not have a significant effect on other parameters (low-density lipoprotein, high-density lipoprotein, free fatty acids, and adiponectin). Only after 16% weight loss, the plasma concentration of free fatty acids, C-reactive protein, and adiponectin improved significantly. Moreover, this study demonstrated that liver and adipose tissue sensitivity improved with a 5% weight reduction and remained stable, whereas muscle insulin sensitivity continued to improve with weight losses of 11% and 16%, respectively [46].

The main advantages of the BIA method in the assessment of body composition are its noninvasiveness, lack of the necessity for highly qualified personnel, short duration, and active involvement of the participants. Furthermore, the repeatability of the BIA test is crucial for observing changes in the body composition of an individual, either in a clinical setting or for research purposes [47]. However, this test also has its limitations. Several factors have been shown to influence the measurement parameters, including body structure, obesity status, body temperature, hydration status, water and electrolyte imbalance, physical activity, and errors while performing the procedure [47]. This study tried to reduce some of these limitations by properly preparing for the examination, such as instructing the participants to fast at least 6 hours before the procedure and ensuring compliance with the procedure during measurements. Apart from the abovementioned limitations, the literature has reported inconsistent results among ethnicities. On the basis of current data, BIA is still not recommended for African American individuals, but studies have shown that it provided valid results in other populations, including Asian populations, by using race-specific equations [48].

### Strengths and Limitations

This study has several strengths. To our knowledge, this study is 1 of 2 studies that were conducted to investigate the health impact of dry IF twice per week. However, although the other study [31] was conducted among overweight older men, our study allowed for higher generalization of the findings as a broader range of age group (19 to 59 years) of participants and both sexes were involved. Two days of voluntary dry fasting per week (on Mondays and Thursdays) is commonly practiced in countries with a predominantly Muslim population, including Malaysia. The positive implications on health demonstrated in our study will encourage people to practice this IF protocol as a weight management method beyond religious obligation. Those who are interested in practicing but are not accustomed to the 13-hour dry fast, such as non-Muslims, must take special precautions to avoid dehydration, lethargy, and electrolyte imbalance. In addition, although the Malaysian Healthy Plate has been introduced since 2016, there is no study that investigates its effectiveness in improving health. Our findings have added new evidence to the body of knowledge and evaluated the outcome of the Malaysian Healthy Plate at the same time.

The main limitation of this study was the impact of the movement control order implemented during the COVID-19 pandemic on the findings of the study and the participants’ compliance with the intervention. As civil servants, many of our participants worked from home during most of the intervention period. Staying at home may limit their physical activity, influence their food intake control, reduce motivation toward weight loss, and expose them to unhealthy eating. The negative consequences of social lockdowns on weight management and weight-related behavior have been reported in several studies [49,50]. Furthermore, the use of FFQ to measure dietary consumption has several limitations. Despite the requirement for good memory and literacy among participants, the food-based FFQ used in our study may also have led to response errors if the participants did not prepare their food and were unaware of the ingredients. Moreover, the dietary intake tends to be underestimated because various seasonings and culinary oils that significantly contribute to energy and nutrient intakes are not considered when calculating dietary intakes [51].

### Conclusions

To the best of our knowledge, this study is the first to introduce a protocol for healthy eating that combines 2-day per week dry IF with the Malaysian Healthy Plate. Our study found that a combined dry IF and HP diet was effective in reducing weight and improving body composition. Although practicing the HP protocol alone has been demonstrated to reduce weight and improve body composition to some extent, the inclusion of a dry IF component is necessary to further amplify these effects. The significant reduction in calorie consumption in both groups validates the efficacy of both dietary protocols as a feasible and practical method for calorie restriction.

## Appendix

Multimedia Appendix 1 Detailed results.

## Abbreviations

BIA: bioelectrical impedance analysis
EM: estimated margin
FFQ: Food Frequency Questionnaire
GEE: generalized estimating equation
HP: healthy plate
IF: intermittent fasting
IFHP: intermittent fasting healthy plate
IPAQ: International Physical Activity Questionnaire
MET: metabolic equivalent of task

## Data Availability

The data sets generated and analyzed during this study are available from the corresponding author upon reasonable request.
