# Supplementary material for: The Effect of a Combined Intermittent Fasting Healthy Plate Intervention on Anthropometric Outcomes and Body Composition Among Adults With Overweight and Obesity: Nonrandomized Controlled Trial
Source: JMIR Form Res. 2024 Apr 10;8:e51542. doi: 10.2196/51542 (PMC11043932; doi:10.2196/51542)
Supplement: Multimedia Appendix 1 [file formative_v8i1e51542_app1.docx]

**Detailed Results**

Independent sample t test for comparison of baseline characteristics of participants between intervention groups.

| Characteristics | *t* | *df* | *P* value |
| --- | --- | --- | --- |
| Age | -1.20 | 175.00 | 0.23 |
| Calorie intake | 0.23 | 147.00 | 0.82 |
| Height | 0.59 | 175.00 | 0.56 |
| Waist circumference | 1.30 | 174.00 | 0.20 |
| Hip circumference | 0.83 | 174.00 | 0.41 |
| Body fat percentage | 0.27 | 173.00 | 0.79 |
| Body fat mass | 1.26 | 173.00 | 0.21 |
| Skeletal muscle mass | 1.53 | 173.00 | 0.13 |
| Visceral fat area | 0.55 | 173.00 | 0.59 |
